# Development and Verification of a Combined Immune- and Metabolism-Related Prognostic Signature for Hepatocellular Carcinoma

**DOI:** 10.3389/fimmu.2022.927635

**Published:** 2022-07-08

**Authors:** Yuanyuan Guo, Jing Yang, Hua Gao, Xin Tian, Xiaojian Zhang, Quancheng Kan

**Affiliations:** ^1^Department of Pharmacy, The First Affiliated Hospital of Zhengzhou University, Zhengzhou, China; ^2^Henan Key Laboratory of Precision Clinical Pharmacy, Zhengzhou University, Zhengzhou, China; ^3^Department of Radiotherapy, The First Affiliated Hospital of Zhengzhou University, Zhengzhou, China

**Keywords:** immune, metabolism, prognosis, hepatocellular carcinoma, TME

## Abstract

Immune escape and metabolic reprogramming are becoming important characteristics of tumor biology, which play critical roles in tumor initiation and progression. However, the integrative analysis of immune and metabolic characteristics for the tumor microenvironment in hepatocellular carcinoma (HCC) remains unclear. Herein, by univariate and least absolute shrinkage and selection operator (LASSO) Cox regression analyses, a prognostic signature associated with tumor microenvironment was established based on five immune- and metabolism-related genes (IMRGs), which was fully verified and evaluated in both internal and external cohorts. The C-index was superior to previously published HCC signatures, indicating the robustness and reliability of IMRGs prognostic signature. A nomogram was built based on IMRGs prognostic signature and various clinical parameters, such as age and T stage. The AUCs of nomogram at 1-, 3-, and 5-year (AUC = 0.829, 0.749, 0.749) were slightly better than that of IMRGs signature (AUC = 0.809, 0.734, 0.711). The relationship of risk score (RS) with immune checkpoint expressions, immunophenoscore (IPS), as well as microsatellite instability (MSI) together accurately predicted the treatment efficacy. Collectively, the IMRGs signature might have the potential to better predict prognostic risk, evaluate immunotherapy efficacy, and help personalize immunotherapy for HCC patients.

## Introduction

Hepatocellular carcinoma (HCC) is one of the most frequent malignant tumors globally with a high morbidity and mortality rate (1), and the 5-year survival rate remains at just 14.1% (2). Currently, rapid progress has been made in immune checkpoint blockade strategies (3–6) (e.g., anti-PD-1, anti-PD-L1/PD-L2, and anti-CTLA-4). Nevertheless, only a small proportion of HCC patients respond positively to and benefit from these therapies (7). The main reason for differences in therapeutic efficacy might be due to the high heterogeneity of the immune microenvironment (8, 9). Consequently, the development and verification of a prognostic signature of the tumor microenvironment (TME) for HCC patients to aid immunotherapy remains of critical importance.

Immune escape (10) and metabolic reprogramming (11) are becoming important characteristics of tumor biology and play key roles in tumor initiation and progression. It is well-known that there is a strong connection between the metabolic system that provides energy and the immune system that defends against pathogens. In addition to defending against pathogens, the immune system is closely related to metabolism (12). Meanwhile, metabolic changes in the tumor microenvironment can suppress the immune system and promote tumor growth (13). Based on solely immune- or metabolism-related genes, prognosis prediction signatures were constructed. For example, Dai et al (14). found that immune-related genes signature could predict outcomes and the effectiveness of immunotherapy in HCC. Yang et al. (15) characterized the molecular features of HCC using the gene expression profile of metabolic genes. He et al. (16) constructed a metabolism-associated gene signature, which could help individualize outcome predictions. However, the liver is not only a metabolic organ, but also an immune organ, which makes the tumor microenvironment of HCC have its specificity in addition to its commonalities with other tumors. The evidences above indicate that it is urgently needed to explore the prognostic significance for the interaction between immune and metabolism.

In this study, a systematic and comprehensive integrative analysis of immune- and metabolism-related genessignature was constructed in HCC, and the prognostic value was analyzed. Moreover, a prognostic nomogram was developed to provide a quantitative analysis tool in order to predict prognostic risk in HCC patients.

## Materials and Methods

### Data Acquisition and Identification of Immune- and Metabolism-Related Genes (IMRGs)

Clinical features and gene expression profilesof HCC samples were downloaded from TCGA (https://portal.gdc.cancer.gov/) and ICGC database (https://dcc.icgc.org/). According to the ratio of 7:3, the TCGA-LIHC participants were randomly assigned to two cohorts: the training cohort (*N* = 262) and the testing cohort (*N* = 108). The clinical characteristics of the two cohorts were summarized in **Supplementary Table S1**. The testing and entire TCGA-LIHC cohorts were used as internal validation sets, while the ICGC-LIRI-JP cohort was treated as an external validation set. A detailed description of the survival follow-up data for ICGC-LIRI-JP cohort could be found in **Supplementary Table S2**. In addition, the genes associated with immunity were acquired from the ImmPort database (17) (https://www.immport.org). The metabolism-related genes were extracted by downloading the “c2.cp.kegg.v7.4.symbols” from MSigDB (Version 7.4).

### Non-Negative Matrix Factorization (NMF) Clustering Algorithm

The “Limma” R package (18) was used for the analysis of the differentially expressed genes (DEGs) in HCC and normal samples. The absolute value of log2 (fold change) > 1 and false discovery rate (FDR) < 0.05 were considered as the criteria to screen the DEGs, from which the differentially expressed IMRGs were extracted. HCC samples were clustered using the NMF method after a univariate Cox analysis was performed. The “nsNMF” algorithm was selected with 100 iterations performed and the number of clusters K was set in the range of 2 to 10.

### Establishment of the Prognostic Signature Based on IMRGs

Univariate Cox regression analysis was conducted to identify the genes related to prognosis in the TCGA training cohort. To establish the prognosis signature, the “glmnet” R package was applied to perform the least absolute shrinkage and selection operator (LASSO) Cox regression analysis. Accordingto the median risk score (RS), the TCGA training cohort was categorized into high- and low-risk groups. The ICGC-LIRI-JP cohort was subsequently analyzed in line with the cutoff value on the TCGA training set.

### Construction of Prognostic Nomogram

On the basis of IMRGs prognostic signature and various clinical parameters, a prognostic nomogram was constructed to predict the survival probability of HCC patients. The predictive performance of the nomogram was evaluated by comparing predicted and actual survival risks. The calibration curves at 1-, 3-, and 5-year were plotted *via* “rms” R package.

### Evaluation of the Response to Immunotherapy

Immunophenoscore (IPS) was calculated using the four main factors, including MHC molecules, immunomodulators, effector cells, and suppressor cells, in theTCIA database (19) (https://tcia.at/home), which was used to predict the therapeutic responses to the four major immune checkpoints (including PD-1 and its two ligands, PD-L1/PD-L2 as well as CTLA-4). Moreover, we analyzed the correlation of IMRGs signature with microsatellite instability (MSI), an indicator used to reflect the efficacy of immunotherapy.

### Statistical Analysis

R software (version 4.0.3) was applied to conduct the statistical analyses with *P* < 0.05 considered statistically significant. The correlation analysis was performed using the Pearson method *via* the “corrplot” R package. The difference between the two groups was compared by Mann–Whitney *U* test. The “survival” R package was used to perform univariate and multivariate Cox hazard regression analyses. The Kaplan–Meier curve was employed to compare the survival difference by log-rank test. The “time ROC” R package was used to conduct the receiver operator characteristic (ROC) curve and the area under the curve (AUC).

## Result

### Identification of HCC Molecular Subtypes Based on NMF Algorithm

**Figure 1:**
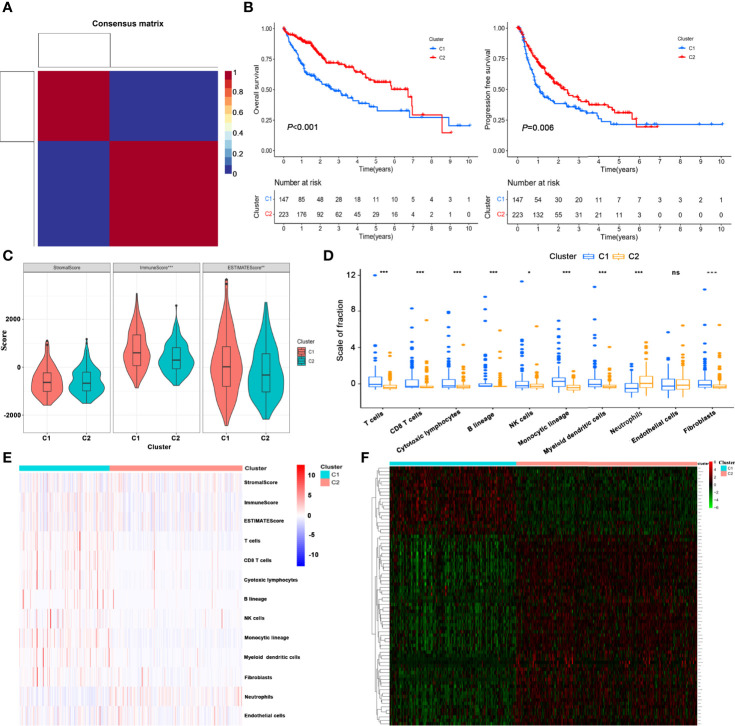
Identification of HCC molecular subtypes based on NMF algorithm. **(A)** Heatmap of nsNMF consensus matrix of *K* = 2. **(B)** Kaplan–Meier curve of overall survival (OS) and progression-free survival (PFS) for HCC subtypes. **(C)** Comparison of immune scores between the two subtypes using estimate algorithm. **(D)** Comparison of immune scores calculated by MCP counter algorithm between the two subtypes. **(E)** Heatmap of the immune scores for ESTIMATE and MCP counter algorithms. **(F)** Unsupervised clustering of immune- and metabolism-related gene expression profiling between the two clusters. ^*^P < 0.05, ^**^P < 0.01, ^***^P < 0.001. ns, no significance.

After filtering and deduplication, a total of 2,715 immune- and metabolism-associated genes (IMRGs) were included. Compared with the normal group, 546 DEGs from IMRGs were observed in HCC samples by further difference analysis, with 441 DEGs upregulation and 105 DEGs downregulation (**Supplementary Table S4**). Then a total number of 257 prognosis-related IMRGs were identified using univariate Cox regression (**Supplementary Table S5**). Afterwards, two molecular subtypes were identified based on the DEGs by the NFM clustering algorithm (**Figure 1A**, **Supplementary Table S6**). The optimal rank value was determined by the indicators of cophenetic, silhouette, and dispersion (**Supplementary Figure S1**). The Kaplan–Meier curve displayed that the overall survival (OS) and progression-free survival (PFS) of cluster 1 were significantly worse than those of cluster 2 (**Figure 1B**). There were significant differences in Immune Score, and ESTIMATE Score between cluster 1 and cluster 2, but not in Stromal Score (**Figure 1C**). Likewise, the proportions of almost 10 immune cells in cluster 1 were higher than that in cluster 2 (**Figures 1D, E**), but the prognosis was worse. It was speculated that the immunosuppressive microenvironment of cluster 1 was composed of exhaust T cells, CD8^+^ T cells, NK cells as well as a high proportion of monocytes-macrophages and neutrophils.

Furthermore, unsupervised clustering of differential expression profiles of immune- and metabolism-related genes between the two clusters were shown in **Figure 1F**. The distribution of clinical parameters between the two molecular subtypes was compared. The survival time of cluster 1 was lower than that of cluster 2, and the mortality rate was significantly higher. The proportions of patients with grade (G3–4), and T stage (T3–4) in cluster 1 were higher than those in cluster 2 (**Supplementary Figure S2**).

### Construction of the IMRGs Prognostic Signature Using LASSO Cox Regression Analysis in TCGA Training Cohort

**Figure 2:**
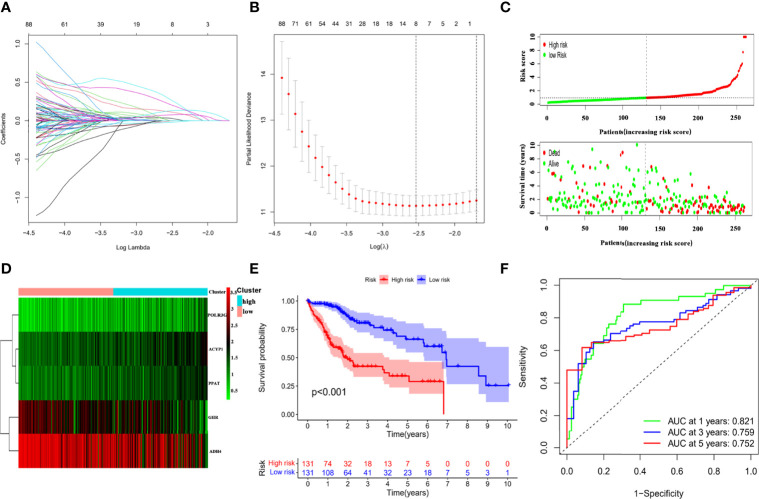
Establishment of the IMRGs prognostic signature using LASSO Cox regression analysis in TCGA training cohort. **(A)** Coefficients of independent variables in LASSO regression. **(B)** The optimal log value of lambda was indicated by the first black dotted line from the left. **(C)** Kaplan–Meier curve analyses for the high-risk group and low-risk group were classified according to the median risk score (RS). **(D)** The ROC curves of the IMRGs prognostic signature at 1-, 3-, and 5-year. **(E)** Distribution of RS and survival status. **(F)** Heatmap of the gene-expression profiles of the IMRGs prognostic signature.

The TCGA-LIHC cohort was randomly divided into two groups in a 7:3 ratio, namely, the training cohort and testing cohort, and no significant differences in clinical features were demonstrated between the two groups (**Supplementary Table S7**). The LASSO Cox regression analysis was used to construct a prognostic prediction model based on the prognosis-related IMRGs in TCGA training cohort. Coefficients of independent variables in LASSO regression were shown in **Figure 2A**. Based on the optimal log value of lambda, we identified 8 genes (**Figure 2B**), among which PPIA and GHR were immune-associated genes, and ACYP1, ADH4, G6PD, POLR3G, PPAT, and UCK2 were metabolism-associated genes. By multivariate Cox regression analysis, a risk score (RS) that represented the comprehensive index of immune and metabolism status for IMRGs signature was calculated by each gene expression multiplied by the corresponding coefficient as follows:


RS=−0.148766×GHR+0.286961×ACYP1−0.089981×ADH4+0.755085×POLR3G+0.593780×PPAT.

The forest plot displayed that the five genes in the risk mode were closely related to prognosis (**Supplementary Figure S3A**). And Wilcoxon test showed that there were significant differences in the expression of five genes in the high- and low-risk groups. The expressions of ACYP1, POLR3G, and PPAT were higher in high-risk group, while the expressions of GHR and ADH4 were higher in low-risk group (**Supplementary Figure S3B**). Then, based on the median of RS, the HCC samples in the TCGA training cohort were divided into high- and low-risk groups to probe the association between the RS and prognosis. Scatter plot depicted the distribution of RS and their relationship to survival outcomes (**Figure 2C**). Heat map presented expression profiles of risk genes in prognostic models for high- and low-risk groups (**Figure 2D**). According to the Kaplan–Meier curve, HCC samples with high RS had a poor prognosis (**Figure 2E**). The AUCs of the prognostic model reached 0.821, 0.759, and 0.752 at 1-, 3-, and 5-year, respectively (**Figure 2F**), which exhibited good prognostic value.

### Internal and External Validation of the IMRGs Prognostic Signature

To further assess the robustness and predictive ability of the IMRGs signature, both internal (TCGA testing cohort and entire TCGA-LIHC cohort) and external validations (ICGC-LIRI-JP cohort) were performed. The RS of validation groups was determined according to the same formula as the TCGA training set. Likewise, the high- and low-risk groups were classified using the same cutoff value as the training group.

**Figure 3:**
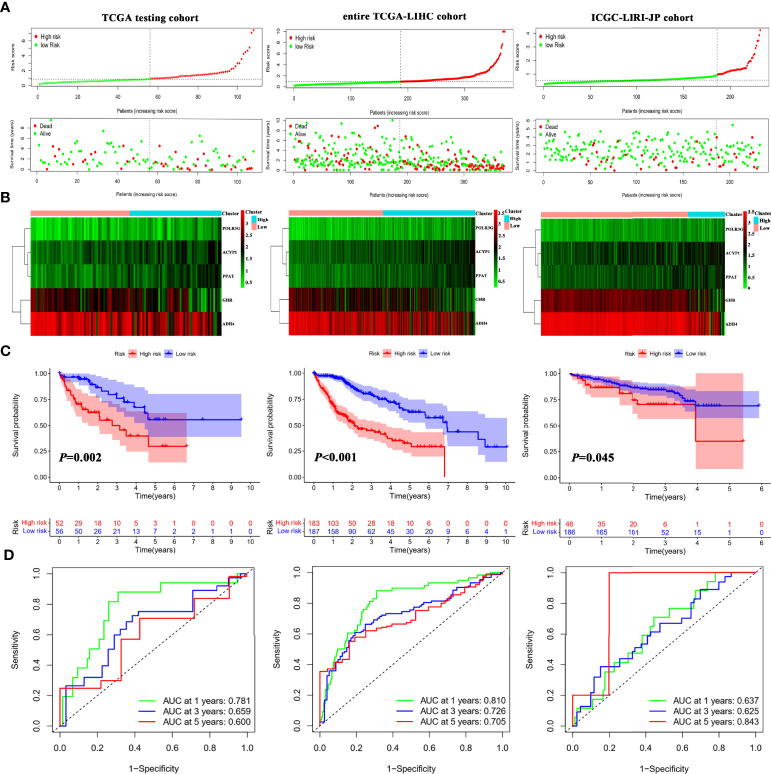
Internal and external validation of the IMRGsprognostic signature. **(A)** Distributionof RS and survival status in internal (TCGA testing and entire TCGA-LIHC) and external (ICGC-LIRI-JP) cohorts. Heatmap of the gene-expression profiles of the IMRGs prognostic signature **(B)**, Kaplan–Meier curve analyses for high-risk group and low-risk group **(C)**, and the ROC curves of the IMRGs prognostic signature at 1-, 3-, and 5-year **(D)** in TCGA testing, entire TCGA-LIHC as well as ICGC-LIRI-JP cohorts.

The distribution of the RS and their associations with survival status were illustrated in **Figure 3A**. **Figure 3B** displayed the heatmaps of gene expression profiles included in prognostic models. Significant prognostic differences were found between the high-and low-riskgroups in the TCGA testing (*P* = 0.002), entire TCGA-LIHC (*P* < 0.001), and ICGC cohorts (*P* = 0.045; **Figure 3C**). Additionally, the AUCs of 1-, 3-, and 5-year survival in the TCGA testing, entire TCGA-LIHC, and ICGC cohorts were shown in **Figure 3D**. These results suggested that the IMRGs signature exhibited high performance in terms of robustness and predictive ability.

### Correlation of theIMRGs Prognostic Signature With Clinical Features

**Figure 4:**
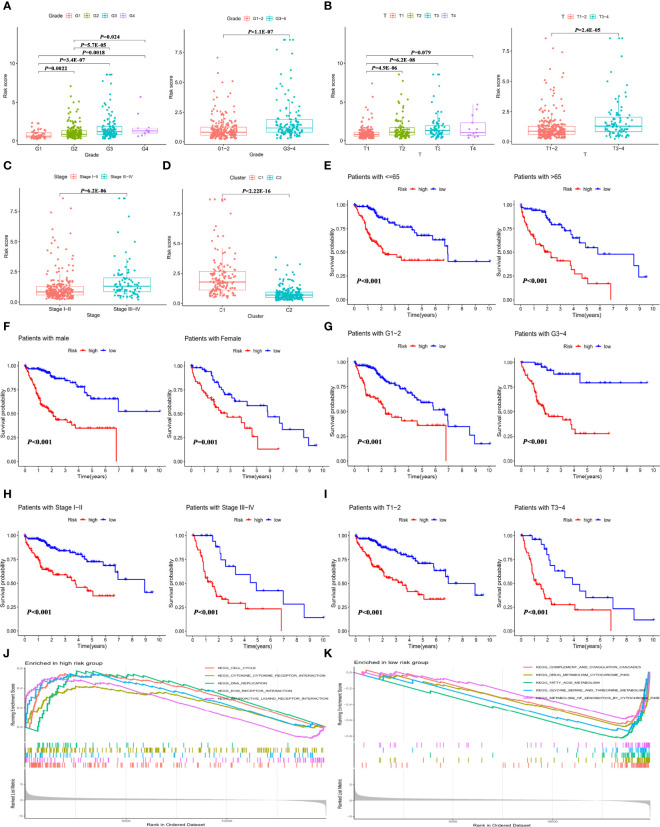
Correlation of the IMRGs prognostic signature with clinical features in the entire TCGA-LIHC cohort. The difference in RS between different clinical features. **(A)** Tumor grade; **(B)** T stage; **(C)** tumor stage; and **(D)** cluster molecular subtypes. Kaplan–Meier curve analyses of OS in clinical subtypes in different clinical subtypes. **(E)** Age ≤65 years and age >65 years; **(F)** gender; **(G)** tumor grade 1–2 and tumor grade 3–4; **(H)** tumor stage I–II and tumor stage III–IV; and **(I)** T stage 1–2 and T stage 3–4. GSEA enrichment analysis in the high-risk group **(J)** and low-risk group **(K)**.

To investigate whether the IMRGs signature correlated with clinical features, the differences in RS were compared in the entire TCGA-LIHC cohort by independent *t* tests. The RS of HCC samples with tumor grade (**Figure 4A**), T stage (**Figure 4B**), and advanced pathological stage (**Figure 4C**) was higher than that of the corresponding early-stage samples. The RS of cluster 1 with a poor prognosis was higher than that of cluster 2 (**Figure 4D**). Based on subgroup analysis, significant differences in prognosis existed between high- and low-risk groups regardless of clinical features such as age (**Figure 4E**), gender (**Figure 4F**), tumor grade (**Figure 4G**), pathological stage (**Figure 4H**), and T stage (**Figure 4I**). These findings indicated that the IMRGs prognostic signature showed good prognostic predictive power according to different clinical characteristics.

Additionally, gene set enrichment analysis (GSEA) for the high-risk (**Figure 4J**) and low-risk (**Figure 4K**) groups of the entire TCGA-LIHC cohort was conducted. The results found that the genes in the low-risk group were significantly enriched in metabolism-related pathways, which were detailed in **Supplementary Table S8**. It can be inferred from the pathway enrichment analysis that alterations in metabolic pathways might lead to different immune status in the high-risk group.

### Comparison of the IMRGs Prognostic Signature With the Published Signatures

**Figure 5:**
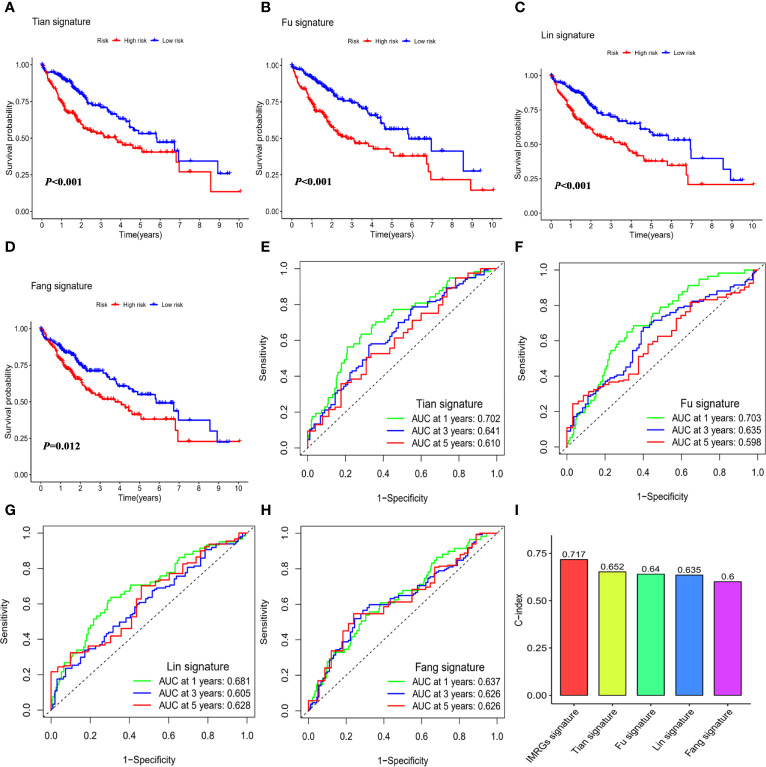
Comparison of the IMRGs prognostic signature with the published signatures. Kaplan–Meier survival curve and ROC curve of five genes signature established by Tian et al. (Tian signature; **A**, **B**), three genes signature established by Fu et al. (Fu signature; **C**, **D**), eight genes signature established by Lin et al. (Lin signature; **E**, **F**), six genes signature established by Fang et al. (Fang signature; **G**, **H**). **(I)** Comparison of C-indexes for the five prognostic models.

To explore whether the immune- and metabolism-associated model had a superior predictive ability, we compared it with four published prognostic models, namely Tian signature (20) (a five-gene model), Fu signature (21) (a three-gene model), Lin signature (22) (an eight-gene model) and Fang signature (23) (a six-gene model). To make the signatures comparable, the same method was applied to calculate and convert the RS of the entire TCGA-LIHC cohort. All the published four signatures were able to categorize the HCC samples into a high-risk group and low-risk group with significantly different outcomes (**Figures 5A–D**). Nevertheless, ROC curve analysis found that the AUCs of the published four signatures were lower than those of our model with AUCs of 0.810, 0.726, and 0.705 for 1-, 3-, and 5-year survival, respectively (**Figures 5E–H**). Furthermore, the C-index was highest in our model at 0.717, followed by Tian signature (C-index = 0.652), Fu signature (C-index = 0.64), Lin signature (C-index = 0.635), and Fang signature (C-index = 0.6; **Figure 5I**). The findings highlighted consistently superior performance of IMRGsprognostic signature.

### Construction of the Nomogram Based on the IMRGs Prognostic Signature and Evaluation of Clinical Significance

**Figure 6:**
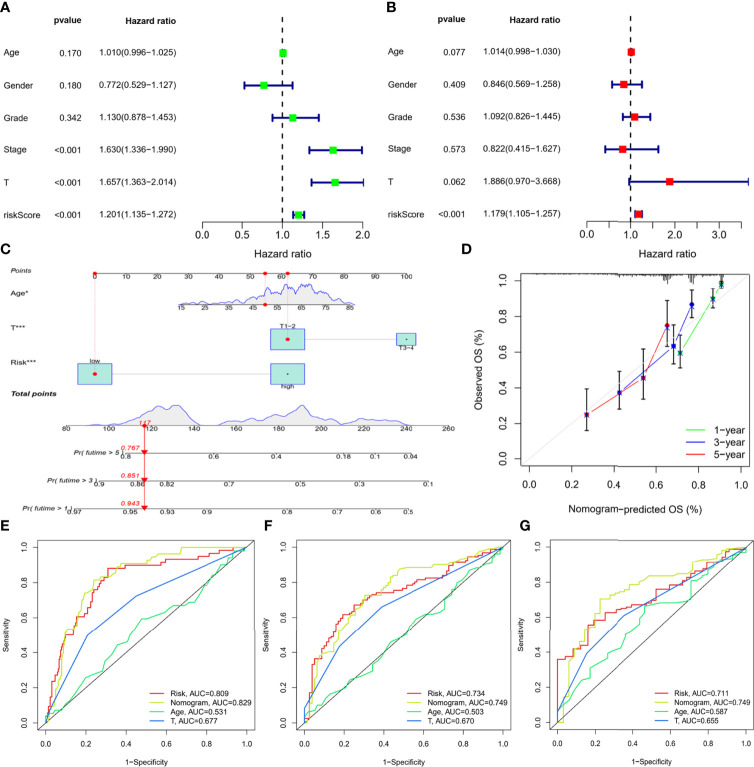
Construction of the nomogram based on the IMRGs prognostic signature and evaluation of clinical significance in the entire TCGA-LIHC cohort. **(A, B)** Univariate and multivariate Cox regression analyses of RS and various clinical features. **(C)** Nomogram for predicting the OS in the entire TCGA-LIHC cohort at 1-, 3-, and 5-year. For each patient, the total score was calculated by adding the points determined by the point scale of each variable. Based on the total points, the bottom scale was used to predict the probability of 1-, 3-, or 5-year survival. The red line exemplified the calculation process and principle of the nomogram. **(D)** Calibration curve for consistency between 1-, 3-, or 5-year nomogram predicted survival and actual survival. **(E–G)** ROC curves of nomograms for 1-year, 3-year, and 5-year survival.

To assess the independence of the IMRGs prognostic signature for clinical application, Cox regression analyses were performed in the entire TCGA-LIHC cohort and ICGC-LIRI-JP cohort. In the entire TCGA-LIHC cohort, significant correlations between RS and prognosis were found in both univariate [hazard ratio (95% CI) = 1.201 (1.135–1.272), *P* < 0.001] (**Figure 6A**) and multivariate regression analyses [hazard ratio (95% CI) = 1.179 (1.105–1.257), *P* < 0.001] (**Figure 6B**). The results were further verified in ICGC-LIRI-JP cohort (**Supplementary Figure S4**), suggesting that the IMRGs prognostic signature exhibited a good clinical predictive value.

Then, three variables with a *P* value less than 0.1 were determined by multiple regression, namely, age, T stage, and RS, and a nomogram was constructed to predict survival risk for individuals (**Figure 6C**). As shown in **Figure 6D**, the calibration curves displayed good consistency between the nomogram-predicted survival and actual survival. Moreover, the AUCs for the nomogram were 0.829, 0.749, and 0.749 at 1-, 3-, and 5-year, which were also higher than the other two variables (**Figures 6E–G**). Thus, the results indicated thatthe nomogram based on the IMRGs prognostic signature showed a significant relation to prognosis and helped predict disease progression.

### Predictive Role of the IMRGs Prognostic Signature in Response to Immunotherapy

**Figure 7:**
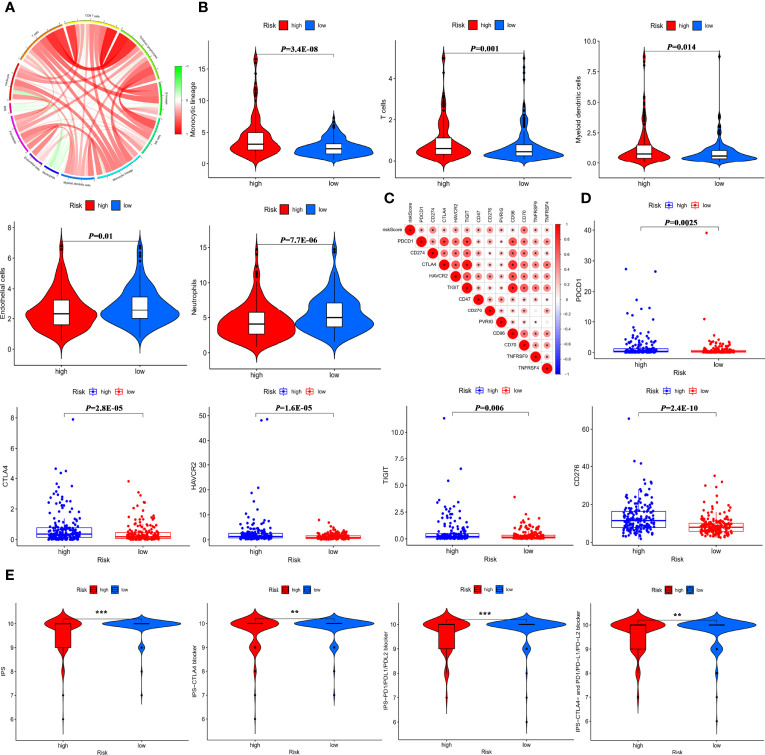
Predictive role of the IMRGs prognosticsignature in response to immunotherapy. **(A)** Correlation of the RS, MSI, and MCP counter immune scores. **(B)** Differences in immune cell infiltration between the high-risk group and low-risk group. **(C)** Correlation of the RS and immune check point expressions. **(D)** Comparison of the difference in immune checkpoint expressions (such as PD-1/PDCD1, CTLA4, TIM-3/HAVCR2, TIGIT, B7-H3/CD276) between high- and low-risk groups. **(E)** Correlation between the RS and four IPS scores related to a single ICI or a combination of ICIs including PD-1, PD-L1/PD-L2, and CTLA-4. ^*^P < 0.05, ^**^P < 0.01, ^***^P < 0.001.

To further seek the effect of the IMRGs prognostic signature on immunotherapy efficacy, correlations between the RS and immune infiltration of TME were analyzed. As shown in **Figure 7A**, the RS was negatively associated with neutrophils, but positively associated with monocytic lineage and myeloid dendritic cells, cytotoxic lymphocytes, and fibroblasts as well MSI (24), a well-established biomarker for predicting immune efficacy. In the low-risk group, the proportions of monocytes, myeloid dendritic cells, and T cells were significantly decreased, while the proportions of neutrophils and endothelial cells were significantly increased (**Figure 7B**). Furthermore, the RS was significantly positively associated with immune checkpoint expressions (**Figure 7C**). There were significant differences in the expression of immune checkpoints such as PD-1/PDCD1, CTLA4, TIM-3/HAVCR2, TIGIT, and B7-H3/CD276 between high- and low-risk groups (**Figure 7D**). Additionally, the relationship was explored between the RS and IPS, excellent indicators in predicting the response to immunotherapy. Significant differences were found between the high-risk and low-risk groups in terms of IPS, IPS-CTLA4, IPS-PD1/PD-L1/PD-L2 blockers, and IPS-CTLA4+PD1/PD-L1/PD-L2 blockers (**Figure 7E**). The findings indicated that the IMRGs prognostic signature could potentially reflect the immune infiltration status and predict the response to immunotherapy.

## Discussion

The advent of the era of immunotherapy has greatly improved outcomes for HCC patients. However, not all patients can get benefit from it, which might be close to the differences in the TME of HCC patients. Given the importance of the interaction between immune and metabolism, it is reasonable to expect that the model would have a good performance in predicting prognosis based on the immune- and metabolism-related genes. To our knowledge, a systematic and comprehensive integrative analysis of immune- and metabolism-associated genes characteristic remains poorly understood in HCC. In this study, an immune- and metabolism-related genes signature was constructed, and the prognostic value was verified. Besides, a nomogram was constructed based on five immune- and metabolism-related genes and clinical features. The clinical significance of the IMRGs prognostic signature was assessed by comparing the immune checkpoint expressions between the high-risk group and low-risk groups and exploring the predictive role in response to immunotherapy.

The entire TCGA-LIHC cohort was categorized into two subtypes using NMF algorithm based on 546 DEGs. Compared with cluster 2, cluster 1 exhibited a poor prognosis, which might be related to the immunosuppressive microenvironment formed by a higher degree of immune infiltration identified by ESTIMATE (25) and MCP counter (26) algorithm. Based on univariate Cox regression and LASSO Cox regression analysis, a prognostic signature of IMRGs consisting of GHR, ACYP1, ADH4, and PPAT was constructed in the TCGA training cohort. Based on the median of RS, the prognostic model was categorized into high- and low-risk groups. Further analysis found that the high-risk group showed more advanced pathological stage, T stage, and tumor grade. Subgroup analysis showed that the prognostic model exhibited good prognostic prediction performance regardless of clinical factors. Besides, the model was validated in internal and external cohorts. The C-index of IMRGs prognostic signature was superior to the four previously reported signatures. All the findings suggested that the immune- and metabolism-related prognostic signature had better prognostic ability.

Growth hormone receptor (GHR), a member of the class I cytokine receptor superfamily, was down-regulation in the high-risk group and was related to chemoresistance, tumor metastasis, and poor prognosis (27–29). Acylphosphatase 1 (ACYP1) involved in the formation of acetic acid from acetyl phosphate, was reported to be related to drug resistance such as imatinib. ACYP1, which was highly expressed in HCC, also was associated with decreased survival time (30). High ACYP1 expression promoted cell survival and apoptosis through the JAK/STAT and PI3K/AKT pathways (31). ADH4, an alcohol dehydrogenase, played critical roles in ethanol metabolism (32). The expression of ADH4 was mediated by miR-148a *via* an AGO1-dependent manner (33) and could be considered as a prognostic biomarker or molecular target for patients with HCC (34, 35). POLR3G, one form of RNA polymerase III, was mainly expressed in stem and cancer cells. Increased gene expression of POLR3G was involved in the proliferation and differentiation of cancer cells and characterized by poor prognosis (34). However, the roles of immune and metabolism-related genes such as GHR, ACYP1, ADH4, POLR3G, and PPAT in the immune environment of HCC were unclear, and further experimental verification was required.

The advent of immunotherapy has provided new ideas for the treatment of HCC, of which immune checkpoint inhibitors (ICIs) have become a potentially effective therapeutic strategy (36–38). The response to ICIs was evaluated by the four scores of IPS, all of which have been shown good performance in predicting the response to immunotherapy efficacy (39). To probe the predictive value of IMRGs prognosticsignature on predicting the response to ICIs, the correlation of RS and IPS was assessed. All the four scores related to a single ICI or a combination of ICIs were higher in the low-risk group, indicating that the IMRGs prognostic signature might have the potential power to predict the immunotherapy efficacy and help personalize immunotherapy for HCC patients. A nomogram is used as a new prognostic tool to improve the accuracy of prognostic prediction (40, 41). A nomogram was constructed by integrating the IMRGs prognostic signature and the clinical parameters identified by univariate and multivariate Cox regression analysis. The results showed that the AUCs of nomogram at 1-, 3-, and 5-year (AUC = 0.829, 0.749, 0.749) were slightly better than that of IMRGs signature (AUC = 0.809, 0.734, 0.711), which further verified that IMRGs prognostic signature established could better predict the risk of prognosis and survival for HCC patients.

There are several strengths in this research as follows: First, the robustness and reliability of IMRGs prognostic signature were evaluated and validated using multiple datasets, including internal and external cohorts. Second, the associations of RS with immune checkpoint expressions, four IPS scores, as well as MSI were comprehensively and deeply explored. Third, a nomogram for quantitative calculation was developed in order to assist with clinical application. Nevertheless, there are still several limitations in this study. For example, The IMRGs prognostic signature and the nomogram were established based on a retrospective study, which needs to be further verified in large multicenter prospective cohorts.

## Conclusion

The IMRGs prognostic signature was constructed based on the integrated analysis of immune- and metabolism-related genes, which could better predict prognostic risk and the response to immunotherapy. We also developed a nomogram for patients with HCC, providing an effective quantitative analysis tool to realize the clinical application of personalized precision therapy.

## Data Availability Statement

The datasets presented in this study can be found in online repositories. The names of the repository/repositories and accession number(s) can be found in the article/**Supplementary Material**.

## Author Contributions

XT, XZ, and QK conceived and directed the completion of the study. HG collected and downloaded the data. YG conducted the data analysis and drafted the manuscript. JY modified the language and revised the manuscript. All authors contributed to the manuscript and approved the submitted version.

## Funding

This study was funded by the Medical Science and Technology Research Plan Joint Construction Project of Henan Province, China (No. LHGJ20210282) and the National Key Research and Development Program of China (No. 2020YFC2008304).

## Conflict of Interest

The authors declare that the research was conducted in the absence of any commercial or financial relationships that could be construed as a potential conflict of interest.

## Publisher’s Note

All claims expressed in this article are solely those of the authors and do not necessarily represent those of their affiliated organizations, or those of the publisher, the editors and the reviewers. Any product that may be evaluated in this article, or claim that may be made by its manufacturer, is not guaranteed or endorsed by the publisher.

## Abbreviations

HCC, hepatocellular carcinoma; TME, tumor microenvironment; MSI, microsatellite instability; IPS, immunophenoscore; AUC, area under the curve; NFM, non-negative matrix factorization; GSEA, gene set enrichment analysis; ROC, receiver operating characteristic.
