# Supplementary figures and images for: Development and Verification of a Combined Immune- and Metabolism-Related Prognostic Signature for Hepatocellular Carcinoma
Source: Front Immunol. 2022 Jul 8;13:927635. doi: 10.3389/fimmu.2022.927635 (PMC9304746; doi:10.3389/fimmu.2022.927635)

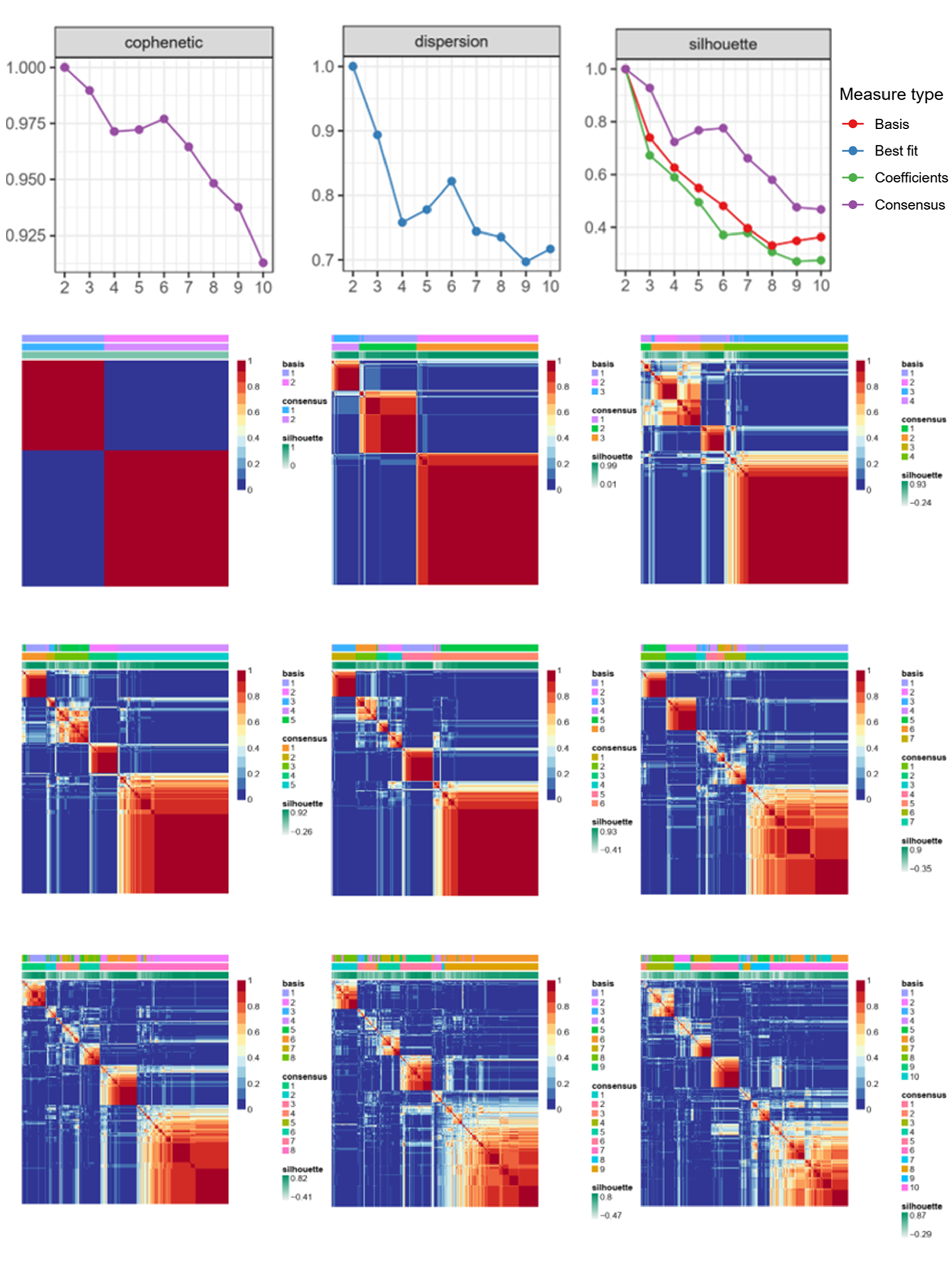

Supplement: Supplementary file 4 [file Image_1.tif]

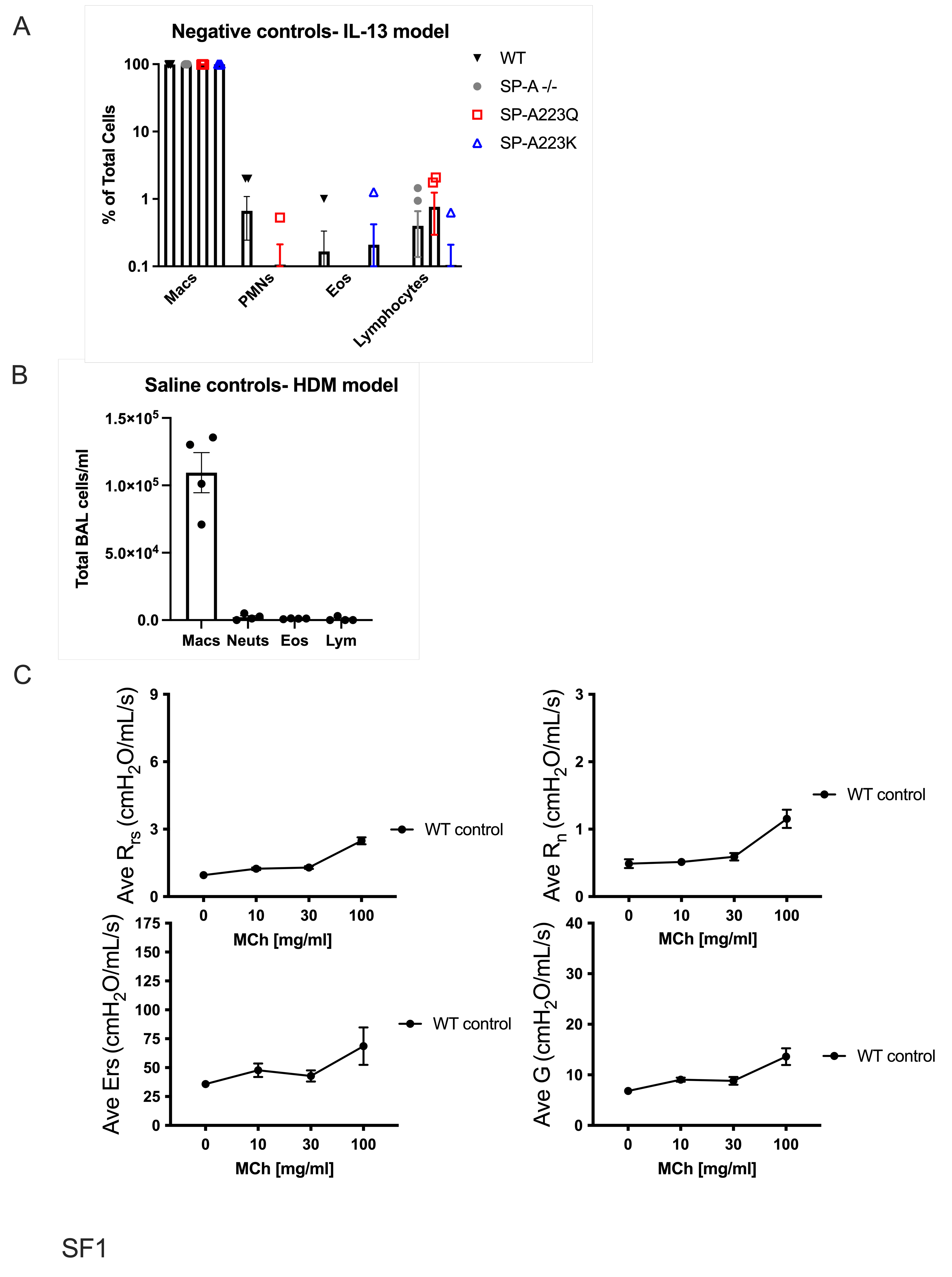

Supplement: Supplementary file 5 [file Image_1.tiff]

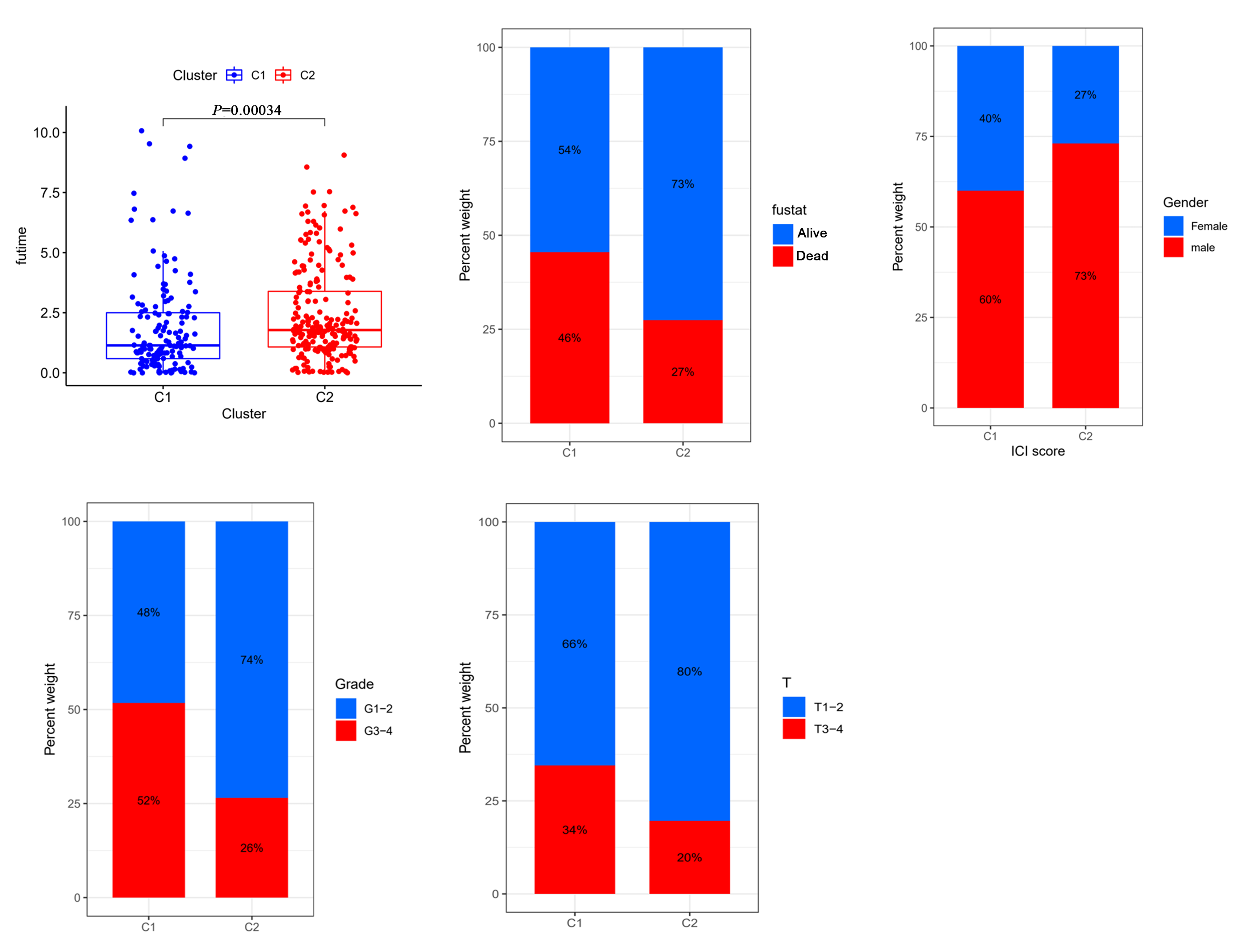

Supplement: Supplementary file 6 [file Image_2.tif]

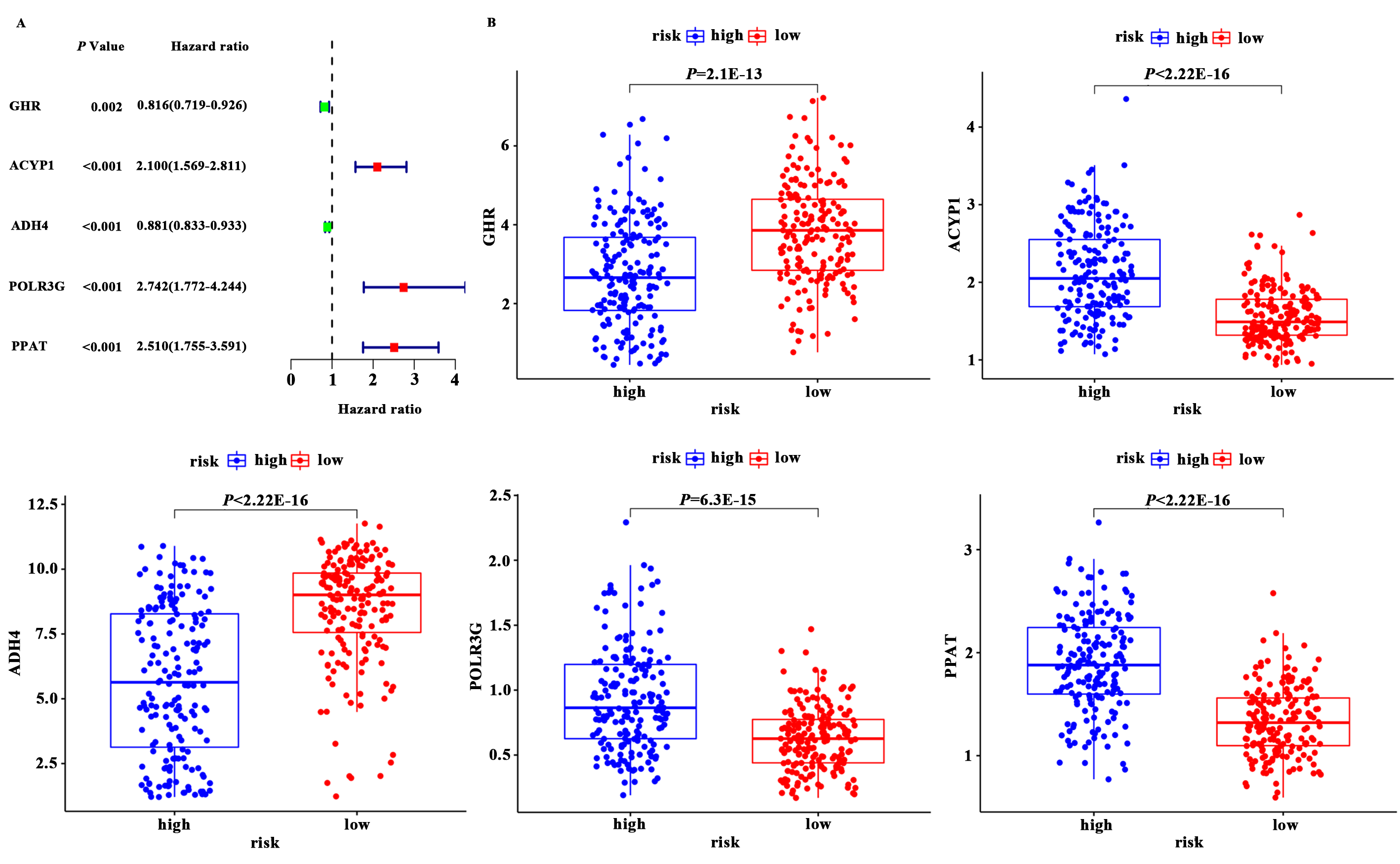

Supplement: Supplementary file 7 [file Image_3.tif]

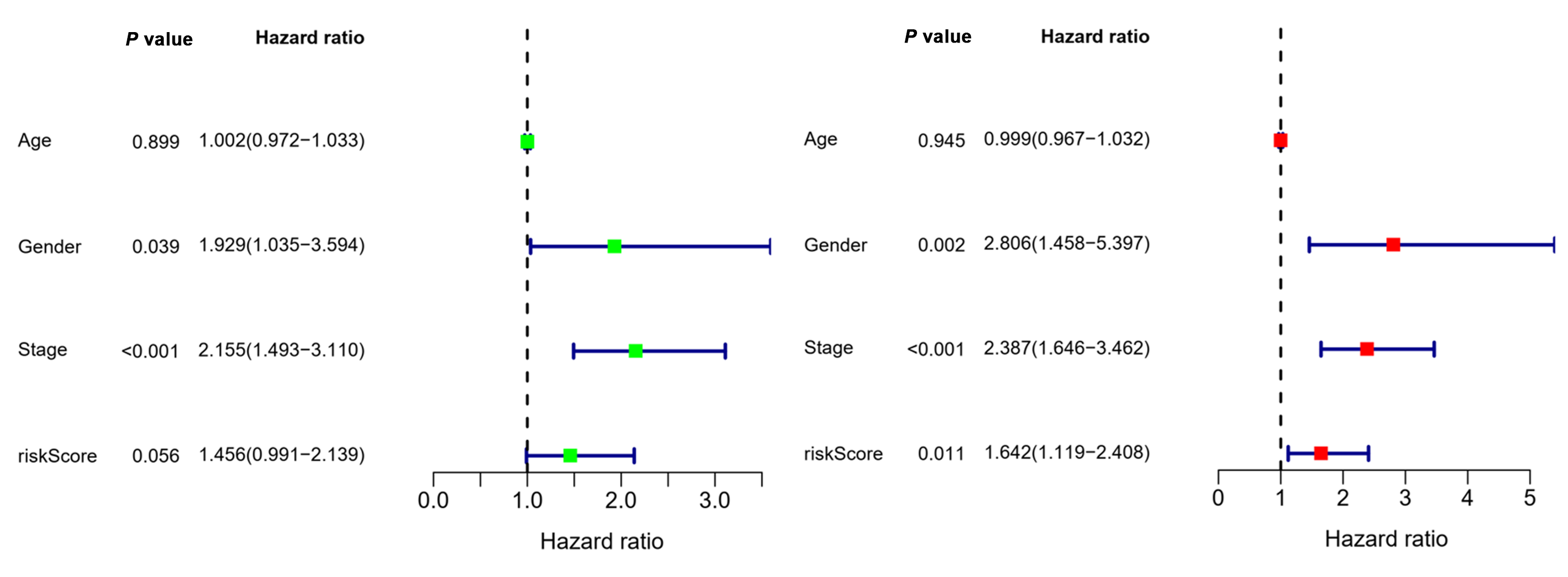

Supplement: Supplementary file 8 [file Image_4.tif]
